# An Evolutionarily Conserved Pathway Essential for Orsay Virus Infection of *Caenorhabditis elegans*

**DOI:** 10.1128/mBio.00940-17

**Published:** 2017-09-05

**Authors:** Hongbing Jiang, Kevin Chen, Luis E. Sandoval, Christian Leung, David Wang

**Affiliations:** Departments of Molecular Microbiology and Pathology & Immunology, Washington University in St. Louis School of Medicine, St. Louis, Missouri, USA; CIML

**Keywords:** *Caenorhabditis elegans*, Orsay virus, TNK2, WASP, *sid-3*, *viro-2*

## Abstract

Many fundamental biological discoveries have been made in *Caenorhabditis elegans*. The discovery of Orsay virus has enabled studies of host-virus interactions in this model organism. To identify host factors critical for Orsay virus infection, we designed a forward genetic screen that utilizes a virally induced green fluorescent protein (GFP) reporter. Following chemical mutagenesis, two Viro (*v*irus *i*nduced *r*eporter *o*ff) mutants that failed to express GFP were mapped to *sid-3*, a nonreceptor tyrosine kinase, and B0280.13 (renamed *viro-2*), an ortholog of human Wiskott-Aldrich syndrome protein (WASP). Both mutants yielded Orsay virus RNA levels comparable to that of the residual input virus, suggesting that they are not permissive for Orsay virus replication. In addition, we demonstrated that both genes affect an early prereplication stage of Orsay virus infection. Furthermore, it is known that the human ortholog of SID-3, activated CDC42-associated kinase (ACK1/TNK2), is capable of phosphorylating human WASP, suggesting that VIRO-2 may be a substrate for SID-3 in *C. elegans*. A targeted RNA interference (RNAi) knockdown screen further identified the *C. elegans* gene *nck-1*, which has a human ortholog that interacts with TNK2 and WASP, as required for Orsay virus infection. Thus, genetic screening in *C. elegans* identified critical roles in virus infection for evolutionarily conserved genes in a known human pathway.

## IMPORTANCE

Orsay virus is the only known virus capable of naturally infecting the model organism *Caenorhabditis elegans*, which shares many evolutionarily conserved genes with humans. We exploited the robust genetic tractability of *C. elegans* to identify three host genes, *sid-3*, *viro-2*, and *nck-1*, which are essential for Orsay virus infection. Mutant animals that lack these three genes are highly defective in viral replication. Strikingly, the human orthologs of these three genes, activated CDC42-associated kinase (TNK2), Wiskott-Aldrich syndrome protein (WASP), and noncatalytic region of tyrosine kinase adaptor protein 1 (NCK1) are part of a known signaling pathway in mammals. These results suggest that TNK2, WASP, and NCK1 may play important roles in mammalian virus infection.

## INTRODUCTION

Viruses are obligate intracellular pathogens that are dependent upon host cell machinery and resources to fulfill their parasitic life cycle. Although great strides have been made in our understanding of many aspects of viral life cycles, there remain many unanswered questions in fundamental virology. Model organisms provide reductionist systems that can circumvent challenges posed by the complexity of mammalian systems. While *Caenorhabditis elegans* has played a critical role in the discoveries of evolutionarily conserved processes such as RNA interference (RNAi) (1), its use in the study of host-virus interactions has been limited by a lack of viruses capable of infecting *C. elegans*. The recent discovery of Orsay virus, the first natural viral pathogen of *C. elegans*, has provided a unique opportunity to exploit this classic model organism to identify host factors critical for virus infection (2, 3).

Orsay virus is a nonenveloped virus with a positive-sense bipartite genome that is most closely related to members of the family *Nodaviridae* (3). The virus genome encodes an RNA-dependent RNA polymerase (RdRp) in the RNA1 segment, while the RNA2 segment encodes the viral capsid protein and a capsid-delta fusion protein of unknown function that is generated by a ribosomal frameshifting mechanism (4). Orsay virus infects primarily *C. elegans* intestinal cells (5) and leads to morphological disruptions of the intestine (3). In the laboratory, experimental infection can be achieved by simply adding Orsay virus to the bacterial lawn upon which *C. elegans* feeds. In terms of antiviral immunity, the roles for RNAi (6, 7) and the ubiquitin proteasome pathway (8) have been identified. Furthermore, there are specific transcriptional responses to Orsay virus infection (8–10). However, little is known about the Orsay virus life cycle. For example, it is not clear what receptor(s) is used by Orsay virus, how it enters the host cells, where the virus establishes replication inside the cells, or how it egresses and spreads. To begin to address these questions, we designed a forward genetic screen to identify in unbiased fashion host factors that are critical to support Orsay virus infection. Here we describe two genes, *sid-3*, a nonreceptor tyrosine kinase, and B0280.13 (renamed *viro-2*), a *C. elegans* ortholog of human Wiskott-Aldrich syndrome proteins (WASPs), which are essential in *C. elegans* for Orsay virus infection. A further targeted RNAi knockdown determined that *nck-1*, a *C. elegans* ortholog of the human noncatalytic region of tyrosine kinase adaptor protein 1 (NCK1), was also essential for Orsay virus infection in *C. elegans*. The human orthologs of these three genes are part of a known kinase signaling pathway (11–13), suggesting that the genes in *C. elegans* likewise form a pathway.

## RESULTS

### A forward genetic screen identified host factors essential for Orsay virus infection in *C. elegans*.

To identify host factors important for Orsay virus infection in *C. elegans*, a forward genetic screen was designed using a previously described transcriptional green fluorescent protein (GFP) reporter strain that is induced by Orsay virus infection (8). This strain, which carries an integrated transgene comprised of the promoter of the *pals-5* gene, a gene strongly induced by Orsay virus infection (8, 10), fused to GFP, was crossed into the virus hypersensitive *rde-1* mutant genetic background to generate a *jyIs8;rde-1* reporter strain that typically leads to GFP expression in >99% of the animals following Orsay virus infection. Using ethane methylsulfonate (EMS) chemical mutagenesis, the goal was to identify mutants that fail to activate GFP expression after Orsay virus infection (Viro [*v*irus *i*nduced *r*eporter *o*ff] mutants). Mutants with this phenotype could be defective in either their ability to support Orsay virus infection or in the pathway that leads to transcriptional activation of GFP expression.

**FIG 1:**
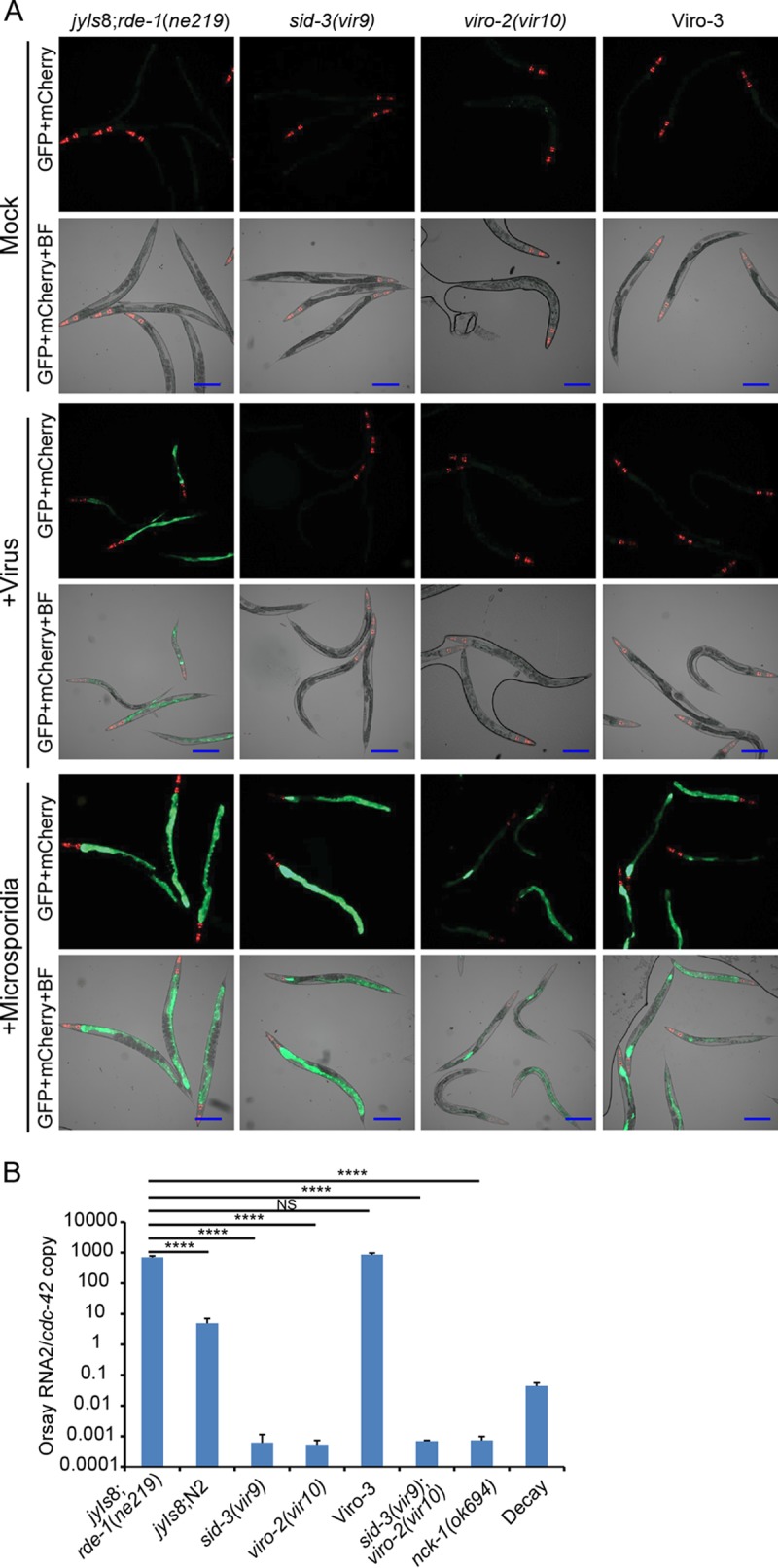
A forward genetic screen identified mutant strains defective in GFP expression and Orsay virus replication. (A) GFP reporter expression patterns of Viro mutants after Orsay virus or *N. parisii* infection. BF, bright-field microscopy. Bars, 200 μm. (B) Orsay virus RNA levels quantified by real-time qRT-PCR. Values are means plus standard deviations (error bars) for three replicate wells. Values that were significantly different (*P* < 0.0005) are indicated by a bar and asterisks (****). Values that were not significantly different (*P* > 0.05) (NS) are indicated.

Approximately 8,000 EMS-mutagenized haploid genomes were screened. Initial Viro hits were rescreened to remove false-positive results (see Fig. S1 in the supplemental material). These strains were bleached and subjected to another round of *de novo* Orsay virus infection, yielding three independent Viro mutants (Fig. 1A). Crossing of the Viro mutants with the unmutagenized reporter strain demonstrated that the Viro phenotype was transmitted as a simple recessive Mendelian trait in all three strains (data not shown). Pairwise mating further demonstrated that these three strains represented three distinct complementation groups, Viro-1, Viro-2, and Viro-3. As described in detail below, a mutation was identified in the *sid-3* gene (*vir9* allele) in Viro-1, and a mutation was identified in the *B0280.13* gene (*vir10* allele) in Viro-2. The B0280.13 gene was subsequently renamed *viro-2*. Hereafter, these mutants are referred to as *sid-3*(*vir9*) and *viro-2*(*vir10*) mutants or strains.

10.1128/mBio.00940-17.1 FIG S1  Scheme of EMS forward genetic screen. (A) Diagram of EMS forward genetic screen using a GFP reporter strain. (B) Work flow for Viro screen using a GFP reporter strain. Download FIG S1, TIF file, 0.6 MB. Copyright © 2017 Jiang et al. 2017 Jiang et al. This content is distributed under the terms of the Creative Commons Attribution 4.0 International license.

Orsay virus RNA levels in these strains were determined by real-time quantitative reverse transcription-PCR (qRT-PCR). The *sid-3*(*vir9*) and *viro-2*(*vir10*) mutants yielded very low levels of Orsay virus RNA (Fig. 1B). These levels were even lower than the amount of Orsay virus RNA detected when input virus was incubated in an empty well for the duration of the experiment and then mixed with a uninfected well of animals immediately prior to RNA extraction (Fig. 1B, bar labeled “Decay”). This control measures the extent of spontaneous degradation of the input virus in the absence of any replication (since no host animals are available to support replication), but it fails to account for any possible negative impact of *C. elegans* (physical movement, feeding, defecation, etc.) on the viral nucleic acid stability on the plate during the course of an authentic infection. Thus, the “decay control” is an upper limit estimate of the amount of residual input Orsay virus RNA. In contrast, Viro-3 Orsay virus RNA levels were similar to that of the unmutagenized parent, suggesting that the defect in Viro-3 affects the pathway necessary for the transcriptional activation of the GFP reporter. In this study, we focused on analysis of *sid-3*(*vir9*) and *viro-2*(*vir10*) mutants.

As the GFP expression in the parental reporter strain can also be induced by infection by *Nematocida parisii*, a microsporidial pathogen of *C. elegans* (8), we challenged *sid-3*(*vir9*), *viro-2*(*vir10*), and Viro-3 mutants with *N. parisii*. All three mutants still induced GFP expression in the intestinal cells, demonstrating that the GFP transgene was still functional and the virus-specific nature of the Viro phenotype (Fig. 1A).

### Mutation in the *sid-3* gene is responsible for Viro-1 phenotypes.

**FIG 2:**
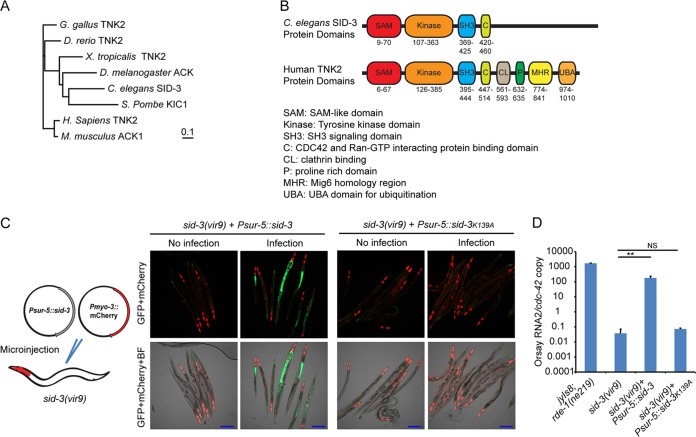
Impact of ectopic expression of SID-3 in *sid-3*(*vir9*) mutant on Orsay virus replication and GFP expression. (A) Neighbor-joining phylogenetic tree of SID-3 orthologs from humans (*Homo sapiens*) and multiple model organisms. The model organisms include *Gallus gallus*, *Danio rerio*, *Xenopus tropicalis*, *Drosophila melanogaster*, *Saccharomyces pombe*, and *Mus musculus*. The bar indicates the amino acid substitution rate (0.1 amino acid substitution per position). (B) Schematic representation of protein domain organization of *C. elegans* SID-3 and human TNK2. (C) Imaging of Orsay virus-infected *sid-3*(*vir9*) mutant that overexpressed wild-type SID-3 or the K139A kinase catalytic domain mutant. BF, bright-field microscopy. Bars, 200 μm. (D) Real-time qRT-PCR of Orsay virus-infected *sid-3*(*vir9*) mutant that overexpressed wild-type SID-3 or the K139A kinase catalytic domain mutant. **, *P* < 0.01; NS, not significant (*P* > 0.05).

To identify the mutation responsible for the lack of GFP expression in the Viro-1 mutant, F2 bulk segregant analysis (14, 15) was performed by outcrossing the strain with the *rde-1*(*ne219*) mutant strain, which is highly susceptible to Orsay virus infection. Approximately 50 F2 progenies that had the Viro phenotype and that did not support Orsay virus replication (data not shown) were identified. The F3 progenies were pooled, sequenced, and analyzed using the CloudMap bioinformatic pipeline (16). Typical mutations introduced by EMS were found in the linked region on chromosome X, including a C-to-T nonsense mutation in the coding region of the *sid-3* (*s*ystemic RNA*i d*efective) gene. This mutation leads to a premature stop after amino acid 911 of the SID-3 protein (Fig. S2). *sid-3* encodes a nonreceptor tyrosine kinase that was originally identified in a genetic screen for animals defective in systemic RNAi (17). In *C. elegans*, *sid-3* is thought to play a role in endosomal import of double-stranded RNA (17). Orthologs of *sid-3* are present in a wide range of organisms (Fig. 2A); the human ortholog ACK1/TNK2 has a wide range of reported substrates for its kinase activity (18, 19) and has been implicated in various cancers (18, 20).

10.1128/mBio.00940-17.2 FIG S2  Schematic representation of *sid-3* and *viro-2* gene structure and their mutant alleles. Download FIG S2, TIF file, 0.2 MB. Copyright © 2017 Jiang et al. 2017 Jiang et al. This content is distributed under the terms of the Creative Commons Attribution 4.0 International license.

**FIG 3:**
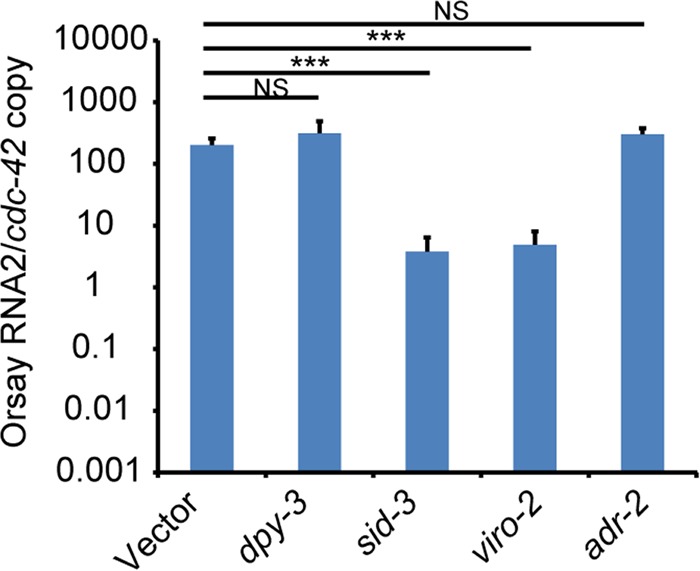
RNAi knockdown of candidate causal genes in *drh-1* mutant strain. Real-time qRT-PCR of Orsay virus RNA levels after feeding RNAi knockdown of individual genes. ***, *P* < 0.005; NS, not significant (*P* > 0.05).

**FIG 4:**
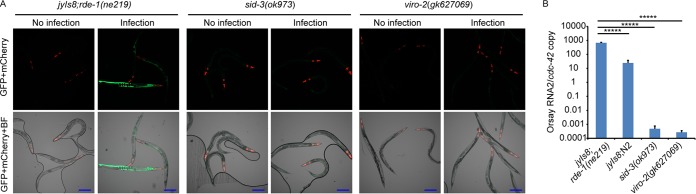
Infection of strains carrying independent mutant alleles of *sid-3* and *viro-2*. (A) GFP expression patterns of reporter strains that carry independent mutant alleles of *sid-3* and *viro-2*. BF, bright-field microscopy. Bars, 200 μm. (B) Quantification of Orsay virus RNA levels by real-time qRT-PCR. *****, *P* < 0.00005.

Several additional lines of evidence demonstrated that *sid-3* was the gene responsible for the observed phenotypes in the Viro-1 strain. First, we performed targeted feeding RNAi knockdown of *sid-3* in the *drh-1* mutant background. While this strain is defective in antiviral RNAi which makes it highly susceptible to Orsay virus infection, it is competent for exogenous feeding RNAi (21). RNAi knockdown of *sid-3* resulted in an approximately 2-log-unit reduction of virus RNA compared to the empty RNAi feeding vector or knockdown of a nonrelated host gene, *dpy-3* (Fig. 3). *sid-3* RNAi in the wild-type N2 background yielded a similar reduction in Orsay virus RNA (Fig. S4). In addition, a strain carrying an independent allele of *sid-3*, VC787 (*ok973*), harboring a >1-kb deletion was obtained from the Caenorhabditis Genetics Center (Fig. S2) and crossed with the *jyIs8;rde-1* reporter strain. This strain phenocopied the *sid-3*(*vir9*) mutant in terms of both GFP expression and Orsay RNA levels (Fig. 4A and B). Similarly, direct infection of *sid-3*(*ok973*) mutant yielded little or no virus replication (Fig. S3). Finally, ectopic expression of wild-type SID-3 from a plasmid driven by the ubiquitous *sur-5* gene promoter rescued the GFP and Orsay virus RNA level phenotypes and partially rescued the virus-induced gene *F26F2.1* (10) expression phenotype (Fig. 2C and D and Fig. S9) as did a fosmid encompassing the *sid-3* locus (data not shown). These data clearly implicated *sid-3* as the gene responsible for the defective GFP expression and reduced Orsay virus RNA levels.

10.1128/mBio.00940-17.3 FIG S3  Infection of CGC strains that carry independent *sid-3* and *viro-2* mutant allele. Orsay virus replication was quantified by real-time qRT-PCR. ****, *P* < 0.0005. Download FIG S3, TIF file, 0.1 MB. Copyright © 2017 Jiang et al. 2017 Jiang et al. This content is distributed under the terms of the Creative Commons Attribution 4.0 International license.

10.1128/mBio.00940-17.4 FIG S4  RNAi knockdown of candidate causal genes in N2 strain. Real-time qRT-PCR of Orsay virus RNA levels after feeding RNAi knockdown of individual genes. **, *P* < 0.01; NS, not significant (*P* > 0.05). Download FIG S4, TIF file, 0.1 MB. Copyright © 2017 Jiang et al. 2017 Jiang et al. This content is distributed under the terms of the Creative Commons Attribution 4.0 International license.

To determine whether the kinase activity of SID-3 was required to complement the Viro phenotype, we ectopically expressed SID-3 carrying a K139A mutation in the catalytic domain that is predicted to abolish its kinase activity in the *sid-3*(*vir9*) mutant. This mutant form of SID-3 had previously failed to rescue the systemic RNAi defect in the *sid-3*(*ok973*) mutant (17). Animals expressing the K139A SID-3 from a *sur-5* promoter failed to rescue either the GFP or RNA phenotype, suggesting that the kinase activity of SID-3 is critical (Fig. 2C and D). In order to rule out any effects of the K139A mutation on SID-3 expression or stability, we expressed C-terminal mCherry fusions of both wild-type and K139A SID-3. As expected, the C-terminal mCherry fusion of the wild-type SID-3, but not the K139A mutant, rescued the GFP and RNA phenotypes (Fig. S5). The level of expression of the K139A SID-3 mutant was higher than the level of expression of wild-type SID-3 as quantified by mCherry expression, demonstrating that the inability of the mutant to rescue the phenotype was not due to lack of protein expression (Fig. S5).

10.1128/mBio.00940-17.5 FIG S5  Impact of ectopic expression of SID-3::mCherry and SID-3K139A::mCherry in *sid-3*(*vir9*) strain on Orsay virus replication and GFP expression. (A) Imaging of Orsay virus-infected strains. Bars, 200 μm. (B) Real-time qRT-PCR of Orsay virus-infected strains. *, *P* < 0.05; NS, not significant (*P* > 0.05). (C) Quantification of SID-3::mCherry and SID-3K139A::mCherry expression through image analysis of mCherry fluorescence intensity. Three individual animals from each representative image were quantified. AU, arbitrary unit; ***, *P* < 0.005. Download FIG S5, TIF file, 1.4 MB. Copyright © 2017 Jiang et al. 2017 Jiang et al. This content is distributed under the terms of the Creative Commons Attribution 4.0 International license.

### Mutation in the *B0280.13* (*viro*-*2*) gene is responsible for Viro-2 phenotypes.

**FIG 5:**
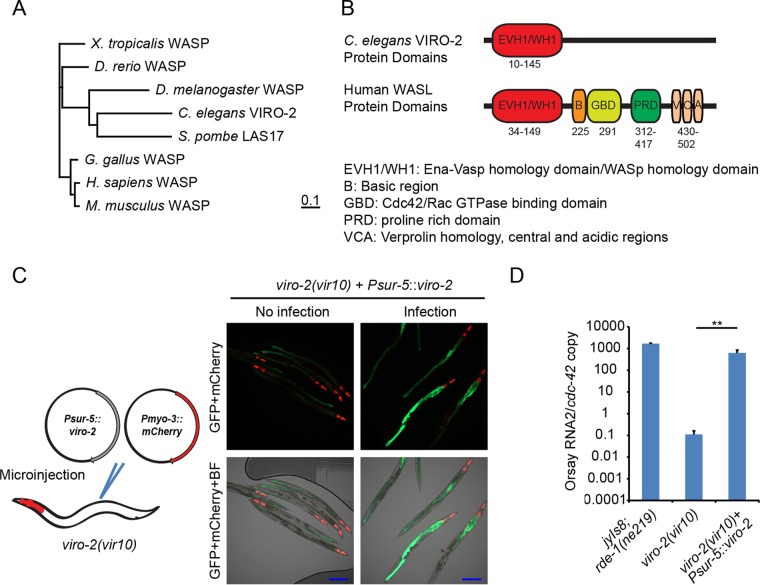
Impact of ectopic expression of VIRO-2 in the *viro-2*(*vir10*) mutant on Orsay virus replication and GFP expression. (A) Neighbor-joining phylogenetic tree of VIRO-2 orthologs from human and multiple model organisms. The bar indicates the amino acid substitution rate (0.1 amino acid substitution per position). (B) Schematic representation of protein domain organization for *C. elegans* VIRO-2 and human N-WASP. (C) GFP expression after ectopic VIRO-2 expression in Viro-2. BF, bright-field microscopy. Bars, 200 μm. (D) Real-time qRT-PCR of Orsay virus-infected *viro-2*(*vir10*) mutant that overexpressed VIRO-2. **, *P* < 0.01.

Two candidate mutations in a linked region of chromosome III were identified in *viro-2*(*vir10*) mutants: a C-to-T mutation which results in a premature stop codon at amino acid position 574 in the *B0280.13* gene (*viro-2*) and a similar mutation at amino acid position 164 in the *adr-2* gene (*a*denosine *d*eaminase acting on *R*NA gene *2*). The *viro-2* gene encodes an ortholog of the human N-WASP (Fig. 5A and B). WASP is an important nucleator of actin polymerization and affects many cellular processes (22). In the context of virus infections, N-WASP is known to be important for egress and spread of vaccinia virus (23, 24). ADR-2 is an RNA-editing enzyme that deaminates adenosines to inosines in double-stranded RNA (dsRNA), and roles for ADR as a proviral factor have been reported (25). To determine which of these genes was responsible for the observed phenotypes, we knocked down both genes using RNAi. RNAi knockdown of *viro-2* yielded a 2-log-unit reduction in Orsay virus RNA levels, while knockdown of *adr-2* gave levels similar to that of the empty RNAi feeding vector, suggesting that the mutation in *viro-2* is responsible for the observed phenotype in this strain (Fig. 3). An independent mutant, *viro-2*(*gk627069*), which harbors a premature stop codon at amino acid position 246 in the C terminus of the *viro-2* gene product, was obtained from the million mutation project (26). After crossing of the strain with the *jyIs8;rde-1* strain, Orsay virus challenge failed to induce GFP (Fig. 4A) and resulted in levels of Orsay RNA similar to those seen in the *viro-2*(*vir10*) strain (Fig. 4B). Overexpression of wild-type VIRO-2 in the *viro-2*(*vir10*) mutant from a microinjected plasmid rescued both the GFP and Orsay virus RNA levels (Fig. 5C and D), as did overexpression of a genomic fragment that contains the *viro-2* gene locus (data not shown). Similar results were obtained with a plasmid expressing a C-terminal mCherry fusion of VIRO-2 (Fig. S6). On the basis of these experiments, we conclude that the mutation in *viro-2* is responsible for the Viro-2 phenotypes.

10.1128/mBio.00940-17.6 FIG S6  Impact of ectopic expression of VIRO-2::mCherry in *viro-2*(*gk627069*) on Orsay virus replication and GFP expression. (A) Imaging of Orsay virus-infected strains. Bars, 200 μm. (B) Real-time qRT-PCR of Orsay virus-infected strains. **, *P* < 0.01. Download FIG S6, TIF file, 1.2 MB. Copyright © 2017 Jiang et al. 2017 Jiang et al. This content is distributed under the terms of the Creative Commons Attribution 4.0 International license.

Furthermore, a double mutant that carried both *sid-3* and *viro-2* gene mutations was generated by crossing the *sid-3*(*vir9*) and *viro-2*(*vir10*) strains. Orsay virus infection of the double mutant showed RNA levels similar to those of *sid-3*(*vir9*) or *viro-2*(*vir10*) single mutant alone (Fig. 1B). As the Orsay virus RNA levels in the single mutants and the double mutant were essentially not detected, no definitive conclusion regarding potential epistasis could be made.

### Orsay virus replication initiated from an endogenous transgene bypasses the requirements for SID-3 and VIRO-2.

**FIG 6:**
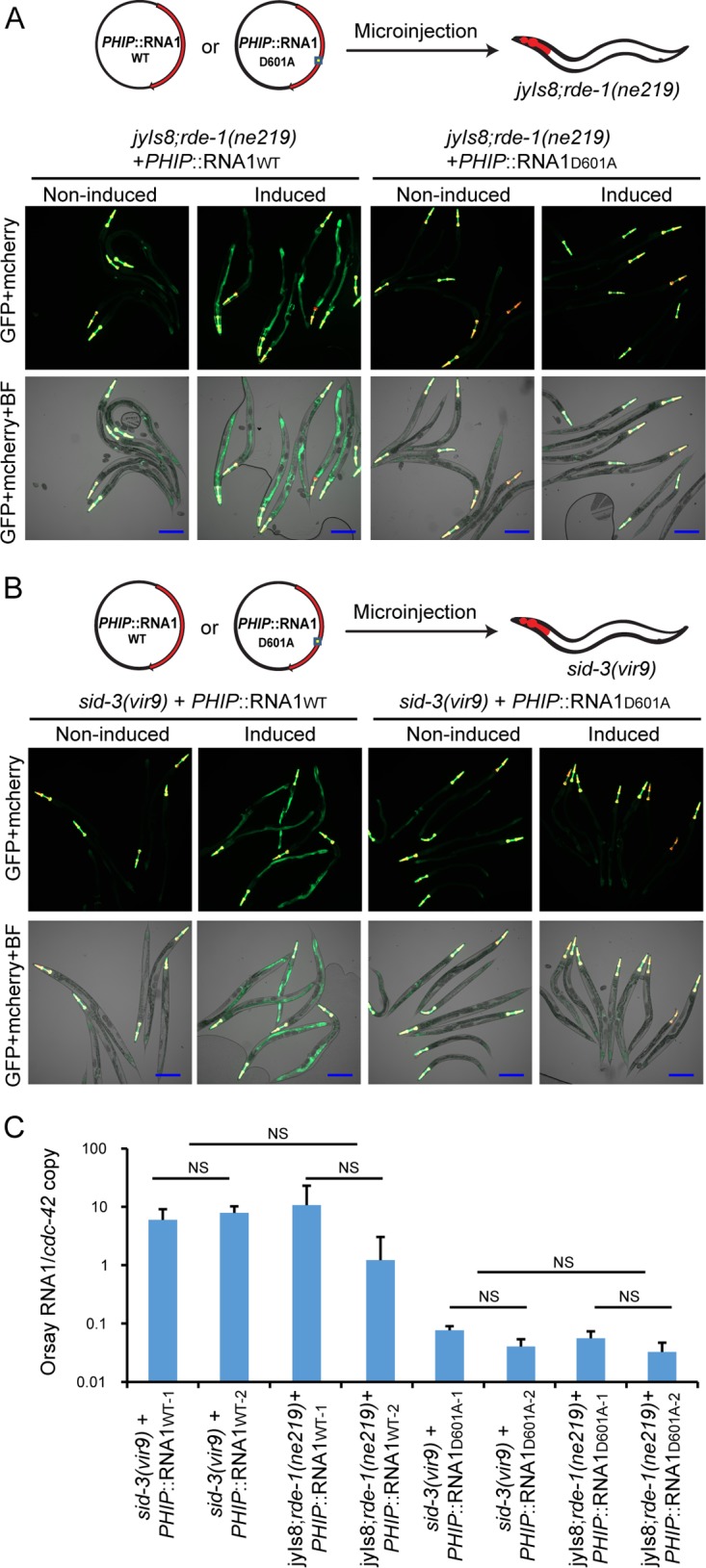
Impact of *sid-3* and *viro-2* on Orsay virus RNA1 transgene-initiated replication. (A) Imaging of heat-induced strains carrying either wild-type or mutant Orsay virus RNA1 in the *jyIs8;rde-1* background. BF, bright-field microscopy. Bars, 200 μm. (B) Imaging of heat-induced strains carrying either wild-type or mutant Orsay virus RNA1 in the *sid-3*(*vir9*) background. Bars, 200 μm. (C) Quantification of Orsay RNA1 replication from heat-induced transgenic *C. elegans*. To control for possible variability in the copy number of the extrachromosomal arrays, two independent lines for each plasmid construct were tested. NS, not significant (*P* > 0.05).

We previously established a two-plasmid cDNA-based reverse genetic system to generate recombinant Orsay virus (27). In this system, Orsay virus RNA1 and RNA2 segments are each expressed under the control of the heat shock promoter, and injection of both plasmids together leads to formation of recombinant Orsay virus. Strains carrying RNA1 alone, which express the RdRp, are competent to support replication of the Orsay virus RNA1 segment as a replicon system. When we microinjected only the plasmid expressing RNA1 into the *jyIs8;rde-1* strain, the resulting strain expressed GFP after heat shock (Fig. 6A). In contrast, microinjection of a plasmid that expresses RNA1 with a D601A mutation in the conserved GDD motif of the RdRp failed to induce GFP expression (Fig. 6A). The former result demonstrates that induction of the plasmid expressing wild-type RNA1 is sufficient to activate GFP expression, while the latter result with the mutant RdRp construct strongly suggests that Orsay virus replication is necessary to activate GFP. To assess the impact of SID-3 on the Orsay virus replicon, we separately microinjected the *PHIP*::RNA1 and *PHIP*::RNA1D601A mutant constructs into the *sid-3*(*vir9*) background to generate extrachromosomal array carrying strains. Heat shock of strain *sid-3*(*vir9*)*;virEx23*[*PHIP*::RNA1WT] [*sid-3*(*vir9*) strain harboring wild-type RNA1] activated GFP expression. Furthermore, increased Orsay RNA1 levels were observed compared to analogous *sid-3*(*vir9*)*;virEx26*[*PHIP*::RNA1D601A] [*sid-3*(*vir9*) strain harboring the D601A replication-incompetent RNA1] demonstrating that Orsay virus RNA1 replication can occur in the absence of SID-3 (Fig. 6C). These results suggest that SID-3 is not required for Orsay virus RNA1 replication initiated from an endogenous transgene.

To confirm these observations in the context of a transgenic system capable of generating recombinant Orsay virus, we generated a stably integrated line (*virIs1;rde-1*) harboring both Orsay virus RNA1 and RNA2. The *virIs1;rde-1* strain was then crossed with the *jyIs8;rde-1* strain to yield the *virIs1;jyIs8;rde-1* strain*.* The *virIs1;jyIs8;rde-1* strain was further crossed with either the *sid-3*(*vir9*) or *viro-2*(*vir10*) strain to generate the *virIs1;jyIs8;rde-1;sid-3*(*vir9*) or *virIs1;jyIs8;rde-1;viro-2*(*vir10*) strain, respectively. When challenged with exogenous Orsay virus, *virIs1;jyIs8;rde-1;sid-3*(*vir9*) and *virIs1;jyIs8;rde-1;viro-2*(*vir10*) strains failed to induce GFP as expected (Fig. S7A). However, heat induction led to expression of GFP, despite the absence of SID-3 or VIRO-2 in these strains (Fig. S7B). These results demonstrate that SID-3 and VIRO-2, while required to induce GFP in response to exogenous virus, are dispensable for induction of GFP in strains that initiate Orsay replication from integrated transgenes. These results suggest that SID-3 and VIRO-2 act at an early stage of the Orsay virus life cycle prior to viral replication.

10.1128/mBio.00940-17.7 FIG S7  Impact of *sid-3* and *viro-2* on Orsay virus transgene-initiated replication. (A) Imaging of exogenous Orsay virus infection of *sid-3*(*vir9*) and *viro-2*(*vir10*) strains carrying integrated Orsay virus genome. Bars, 200 μm. (B) Imaging after heat induction of *sid-3*(*vir9*) and *viro-2*(*vir10*) strains carrying integrated Orsay genome. Bars, 200 μm. Download FIG S7, TIF file, 1 MB. Copyright © 2017 Jiang et al. 2017 Jiang et al. This content is distributed under the terms of the Creative Commons Attribution 4.0 International license.

### SID-3 and VIRO-2 colocalize to the apical side of the intestine.

**FIG 7:**
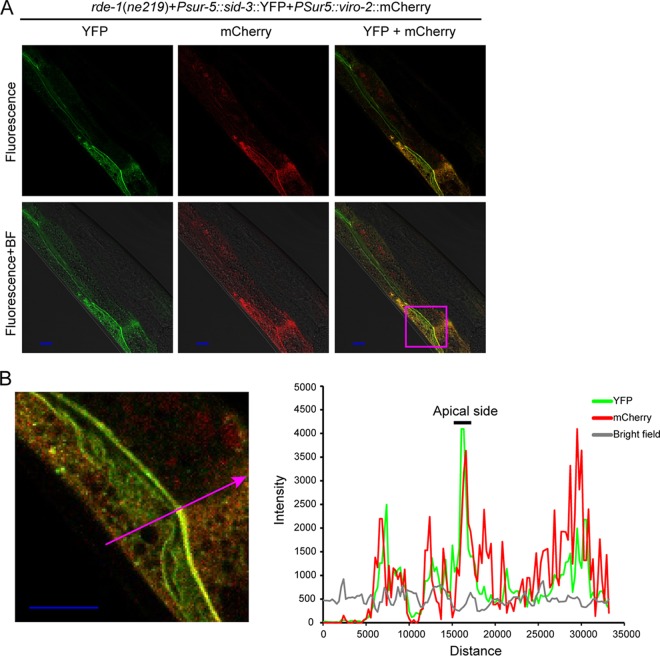
SID-3 and VIRO-2 colocalize in transgenic strains. (A and B) Confocal microscopy of a representative animal of the* rde-1*(*ne219*) strain (*sid-3*::YFP and *viro-2*::mCherry fusion proteins driven by the universal *sur-5* gene promoter) 48 h after Orsay virus infection. BF, bright-field microscopy. Bars, 10 μm. The zoomed-in area shown in the micrograph in panel B is indicated by the magenta box in panel A. The graph in panel B shows fluorescence intensity (in AU [arbitrary unit]) on the *y* axis and distance (in nm) on the *x* axis. The graph shows fluorescence intensity profiling of the position marked by the magenta arrow. The intestinal apical side is indicated in the graph by a black bar.

The human orthologs of SID-3 and VIRO-2 are reported to be a kinase-substrate pair through *in vitro* biochemical studies (19). However, to date, there is no data as to whether SID-3 phosphorylates VIRO-2 in *C. elegans*. Since kinases and their direct substrates must colocalize at some point in time, we asked whether we could detect colocalization of SID-3 and VIRO-2. To this end, we generated strains that express SID-3 C-terminally fused with yellow fluorescent protein (YFP) and VIRO-2 C-terminally fused with mCherry, each driven by the universal *sur-5* gene promoter. Both proteins were detected in somatic cells with stronger expression observed in the intestinal cells. Both tagged proteins were functional, as they rescued the Orsay virus RNA level phenotype when coinjected into the *sid-3*(*vir9*)*;viro-2*(*vir10*) double mutant (data not shown). Colocalization of these two proteins, especially along the apical side of the intestine, was observed in transgenic strains that were infected by Orsay virus (Fig. 7A and B and Fig. S8).

10.1128/mBio.00940-17.8 FIG S8  SID-3 and VIRO-2 colocalize in transgenic strains. (A and B) Confocal microscopy of *rde-1*(*ne219*) strain (SID-3::YFP and VIRO-2::mCherry fusion proteins driven by the universal *sur-5* gene promoter) at 48 h after Orsay virus infection. Bars, 10 μm. The zoomed-in area is indicated by the magenta box in panel A. Graph showed fluorescence intensity profiling of the arrow marked position. The intestinal apical membrane is indicated by a black bar on top. BF, bright-field microscopy. (C) Additional confocal microscopy images of *rde-1*(*ne219*) strain (*sid-3*::YFP and *viro-2*::mCherry fusion proteins driven by the universal *sur-5* gene promoter) with Orsay virus infection. BF, bright-field microscopy. Bars, 10 μm. Download FIG S8, TIF file, 2.7 MB. Copyright © 2017 Jiang et al. 2017 Jiang et al. This content is distributed under the terms of the Creative Commons Attribution 4.0 International license.

### RNAi knockdown defines a role for *nck-1* in Orsay virus infection.

**FIG 8:**
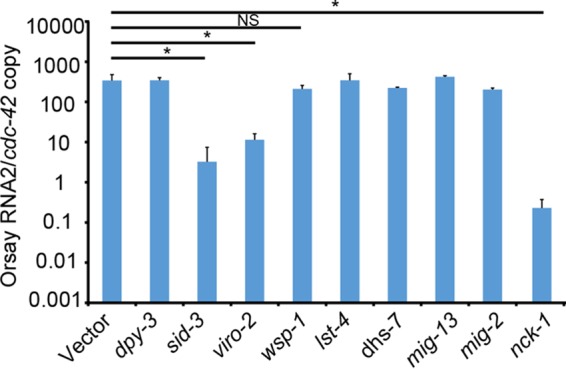
RNAi knockdown of candidate genes. Real-time qRT-PCR of Orsay virus RNA levels after feeding RNAi knockdown of individual genes. *, *P* < 0.05; NS, not significant (*P* > 0.05).

Little is known about *C. elegans* proteins that interact with SID-3 or VIRO-2. In humans, multiple interacting partners of TNK2 and WASP have been defined (12, 13, 28–30). In *C. elegans*, *wsp-1* is another WASP ortholog, and several proteins that interact with *wsp-1* have been identified (31, 32). To determine whether *wsp-1*, the *C. elegans* orthologs of genes that interact with TNK2 (*lst-1*, *dhs-7*, and *nck-1*), or *C. elegans* genes that interact with WSP-1 (*mig-13*, *mig-2*, and *nck-1*) affect Orsay virus infection, we performed RNAi knockdown and evaluated Orsay virus replication. Of these tested genes, *nck-1*, the *C. elegans* ortholog of human NCK1, yielded an approximately 3-log-unit reduction in Orsay virus RNA. These RNA levels are even lower than those observed by RNAi knockdown of *sid-3* and *viro-2* (Fig. 8), which may reflect differences in the relative efficiency of knockdown of the genes. To validate the RNAi result, a strain carrying a mutant allele of *nck-1* (*ok694*) was crossed with a *jyIs8;rde-1* strain and yielded virus levels similar to those of *sid-3*(*vir9*) and *viro-2*(*vir10*) mutant strains when infected by Orsay virus (Fig. 1B). In humans, NCK1 is known to directly bind to both TNK2 (12) and WASP (13), and it is known to play a role in vaccinia virus egress and spreading (24). Together, these data suggest that *sid-3*, *viro-2*, and *nck-1* function in a pathway that plays a critical role in Orsay virus infection in *C. elegans*.

## DISCUSSION

In this study, we identified two genes that are important for Orsay virus infection of *C. elegans* from a small-scale (~8,000 haploid genomes) EMS screen. A third host gene was subsequently identified by additional targeted RNAi screening. As the current EMS screen is far from saturation, additional EMS screening is likely to identify more host factors that play critical roles in Orsay virus infection. From a technical perspective, the use of a GFP reporter renders the screen essentially binary, thereby facilitating large-scale screening.

Neither *sid-3* nor *viro-2* has been extensively studied in *C. elegans*. To date, there is only one published study on *sid-3* that defined a role in endosomal trafficking in the context of systemic RNAi (17) and no explicit published study of *viro-2*. Despite the paucity of knowledge regarding these two genes, much can be inferred from studies of mammals and their orthologs. The human ortholog of *sid-3*, TNK2, has many functions, and multiple substrates for its kinase activity have been identified including N-WASP, clathrin, and androgen receptor (12, 18, 19, 33). The identification of *sid-3* in this screen suggests a possible role for its human ortholog, TNK2, in virus infection. While there are no publications thus far that have focused explicitly on the role of TNK2 in the context of virus infection, it has been identified as a hit in multiple large-scale small interfering RNA (siRNA) screens for influenza A virus (IAV), hepatitis C virus (HCV), and vesicular stomatitis virus (VSV) (34–37). Thus, an unbiased screen of *C. elegans* has identified an evolutionarily conserved gene that may impact virus infection in a range of hosts.

Orsay virus RNA levels in the strains with the two *sid-3* alleles, *vir9* and *ok973*, were 5 to 6 log units lower than those detected in the parental strain [*jyIs8*;*rde-1*(*ne219*)] (Fig. 1B and 4B). Given that the observed RNA levels are lower than the decay, they may represent either very low levels of Orsay virus replication or more likely, simply residual input Orsay virus. On the basis of our observations, we hypothesize that *sid-3* is essential for Orsay virus infection at an early step of the life cycle. The fact that Orsay virus replication initiated from an integrated transgene can still activate GFP expression in the absence of *sid-3* demonstrates that viral replication itself does not formally require *sid-3*; thus, it is likely that *sid-3* acts at a stage preceding virus replication. Furthermore, several of the siRNA screens that identified TNK2 as a host factor important for mammalian virus were specifically designed to detect only genes important for early steps of viral life cycles (34, 36, 38, 39). Collectively, these data suggest that SID-3 (and TNK2) may play roles in early stages such as viral entry; since TNK2 is known to bind directly to clathrin (12), one possibility is that TNK2 and/or SID-3 could modulate clathrin-mediated endocytosis.

While mutations in *viro-2* phenocopy mutations in *sid-3* in terms of both GFP phenotype and Orsay virus RNA levels, the genes encode very different proteins. *viro-2* is one of four *C. elegans* orthologs (along with B0280.2, *wsp-1*, and C31C9.6) of the two human WASP proteins (WASP and N-WASP). VIRO-2 shares an EVH1/WH1 domain with human WASP proteins (Fig. 5B). Wiskott-Aldrich syndrome is characterized by immune dysregulation in humans (40, 41) caused by a deficiency of WASP, which is expressed primarily in hematopoietic cell lineages. N-WASP is ubiquitously expressed in multiple tissue types. WASP proteins are cytoplasmic with several conserved domains. Of the conserved domains, the C-terminal VCA (*v*erprolin homology, *c*entral and *a*cidic regions) domain is involved in regulation of actin polymerization. Normally, the VCA domain of WASP binds to its central GBD (G protein binding domain) and renders this protein inactive. Phosphorylation by a cytosolic kinase combined with either GTP-activated CDC42 or SH2/SH3 domain-containing protein binding synergistically activates N-WASP. This leads to the recruitment of the Arp2/3 complex through its VCA domain initiating actin polymerization and branching (22, 41). There is clear data in human cells demonstrating that vaccinia virus egress and spread are dependent on N-WASP (23, 24). In addition, N-WASP was recently found to play a role in the entry of Lassa virus (42). Given the known ability of WASP proteins to regulate actin polymerization and the need for actin remodeling in many facets of endosomal trafficking (22), a similar role for VIRO-2 in modulating some aspect of endosomal trafficking of Orsay virus is quite plausible, although it is not yet known whether VIRO-2 impacts actin.

Strikingly, the human orthologs of the two *C. elegans* genes identified in this genetic screen have been demonstrated *in vitro* to be part of a kinase-substrate pathway (19). This finding underscores the power of genetic analysis in model organisms and strongly suggests that *sid-3* and *viro-2* may function in a common pathway in *C. elegans*. Our experiments suggest that the kinase activity of SID-3 is important for its ability to promote Orsay virus infection. It is possible that SID-3 directly phosphorylates VIRO-2 much as TNK2 can phosphorylate N-WASP; alternatively, SID-3 could be part of a regulatory cascade and phosphorylate another protein in a pathway that ultimately leads to VIRO-2 phosphorylation. The SID-3 kinase activity could also have other substrates unrelated to VIRO-2 that impact aspects of the Orsay virus life cycle. For example, a link between *sid-3* and *sta-1*, the *C. elegans* ortholog of mammalian Stat genes, has been reported (43). We observed partial colocalization of ectopically expressed SID-3 and VIRO-2 supporting the possibility that they directly interact. Furthermore, RNAi knockdown of the *C. elegans* ortholog of NCK1, a known interacting partner of both human TNK2 and N-WASP, reduced Orsay virus replication to levels similar to that observed with knockdown of *viro-2* or *sid-3*. Thus, we identified *C. elegans* orthologs of multiple human genes that belong to a defined pathway as critical for Orsay virus infection. The studies herein reaffirm the tremendous value and potential of model organism studies to elucidate novel mechanisms that are broadly important across host species.

## MATERIALS AND METHODS

### *C. elegans* culture and maintenance.

*C.  elegans* strains N2, WM27 *rde-1*(*ne219*), RB2519 *drh-1*(*ok3495*), VC787 *sid-3*(*ok973*), RB860 *nck-1* (*ok694*), and VC40417 *viro-2*(*gk627069*) from the million mutation project (26) were obtained from the Caenorhabditis Genetics Center (CGC) and maintained under standard lab culture conditions unless otherwise specified. In brief, animals were fed *Escherichia coli* OP50 on nematode growth medium (NGM) plates in a 20°C incubator and moved every 3 days to a new NGM plate seeded with *E*. *coli* OP50.

### *C. elegans* genetics and crossing.

The reporter strain ERT54 *jyIs*8 [*Ppals-5*::GFP; Pmyo-2::mCherry] (8) was kindly provided by Emily Troemel (University of California, San Diego) and further crossed with the WM27 *rde-1*(*ne219*) strain to generate strain WUM31 *jyIs8* [*Ppals-5*::GFP; Pmyo-2::mCherry];*rde-1*(*ne219*). Strains VC787 *sid-3*(*ok973*) and strain VC40417 *viro-2*(*gk627069*) were crossed with the WUM31 *jyIs8;rde-1* strain, yielding strain WUM57 *jyIs8;rde-1;sid-3*(*ok973*) and strain WUM58 *jyIs8;rde-1;viro-2*(*gk627069*), respectively. The *sid-3*(*vir9*) and *viro-2*(*vir10*) strains were crossed with the WUM28 *virIs1;rde-1* strain, yielding WUM59 *virIs1;jyIs8;rde-1;sid-3*(*vir9*) and WUM60 *virIs1;jyIs8;rde-1;viro-2*(*vir10*), respectively (Table 1).

**TABLE 1:** Transgenic strains used in this study

Lab designation	Strain name	Relevant genotype
WM27	*rde-1*(*ne219*)	[ *rde-1*(*ne219*)V]
ERT54	*jyIs8*	*jyIs8*[*Ppals-5*::GFP; *Pmyo-2*::mCherry]
WUM28	*virIs1;rde-1*(*ne219*)	*virIs1*[*PHIP*::OrsayRNA1;*PHIP*::OrsayRNA2;*Pmyo-2*::YFP; *rde-1*(*ne219*) V]
WUM29	*virIs1;jyIs8;rde-1*(*ne219*)	{*virIs1*[*PHIP*::OrsayRNA1;*PHIP*::OrsayRNA2; *Pmyo-2*::YFP]; *jyIs8*[*Ppals-5*::GFP; *Pmyo-2*::mCherry]; *rde-1*(*ne219*) V}
WUM31	*jyIs8;rde-1*(*ne219*)	{*jyIs8*[*Ppals-5*::GFP;*Pmyo-2*::mCherry]; *rde-1*(*ne219*) V}
WUM32	*jyIs8;rde-1*(*ne219*)*; virEx20*[*PHIP*::RNA1WT-1]	{*virEx20*[*PHIP*::OrsayRNA1WT-1; *Pmyo-2*::YFP]; *jyIs8*[*Ppals-5*::GFP; *Pmyo-2*::mCherry];*rde-1*(*ne219*) V}
WUM33	*jyIs8;rde-1*(*ne219*)*; virEx21*[*PHIP*::RNA1D601A-1]	{*virEx21*[*PHIP*::OrsayRNA1D601A-1; *Pmyo-2*::YFP]; *jyIs8*[*Ppals-5*::GFP; *Pmyo-2*::mCherry];*rde-1*(*ne219*) V}
WUM45	*sid-3*(*vir9*)	{*jyIs8*[*Ppals-5*::GFP; *Pmyo-2*::mCherry]; *rde-1*(*ne219*) V; *sid-3*(*vir9*)X}
WUM46	*viro-2*(*vir10*)	{*jyIs8*[*Ppals-5*::GFP; *Pmyo-2*::mCherry]; *rde-1*(*ne219*) V; *viro-2*(*vir10*)III}
WUM47	Viro-3	Not assigned
WUM51	*sid-3*(*vir9*)*; virEx23*[*PHIP*::RNA1WT-1]	{*virEx23*[*PHIP*::OrsayRNA1;*Pmyo-2*::YFP]; *jyIs8*[*Ppals-5*::GFP; *Pmyo-2*::mCherry]; *rde-1*(*ne219*) V; *sid-3*(*vir9*)X}
WUM52	*sid-3*(*vir9*)*; virEx24*[*PHIP*::RNA1WT-2]	{*virEx24*[*PHIP*::OrsayRNA1;*Pmyo-2*::YFP]; *jyIs8*[*Ppals-5*::GFP; *Pmyo-2*::mCherry]; *rde-1*(*ne219*) V; *sid-3*(*vir9*)X}
WUM54	*sid-3*(*vir9*)*; virEx26*[*PHIP*::RNA1D601A-1]	{*virEx26*[*PHIP*::OrsayRNA1D601A;*Pmyo-2*::YFP]; *jyIs8*[*Ppals-5*::GFP; *Pmyo-2*::mCherry]; *rde-1*(*ne219*)V; *sid-3*(*vir9*)X}
WUM55	*sid-3*(*vir9*)*;virEx27*[*PHIP*::RNA1D601A-2]	{*virEx27*[*PHIP*::OrsayRNA1D601A;*Pmyo-2*::YFP]; *jyIs8*[*Ppals-5*::GFP; *Pmyo-2*::mCherry]; *rde-1*(*ne219*)V; *sid-3*(*vir9*)X}
WUM57	*sid-3*(*ok973*)	{*jyIs8*[*Ppals-5*::GFP; *Pmyo-2*::mCherry]; *rde-1*(*ne219*) V; *sid-3*(*ok973*)X}
WUM58	*viro-2*(*gk627069*)	{*jyIs8*[*Ppals-5*::GFP; *Pmyo-2*::mCherry]; *rde-1*(*ne219*) V; *viro-2*(*gk627069*) III}
WUM59	*virIs1;jyis8;rde-1*(*ne219*)*;sid-3*(*vir9*)	{*virIs1*[*PHIP*::OrsayRNA1;*PHIP*::OrsayRNA2; *Pmyo-2*::YFP]; *jyIs8*[*Ppals-5*::GFP; *Pmyo-2*::mCherry]; *rde-1*(ne219) V;*sid-3*(*vir9*)X}
WUM60	*virIs1;jyis8;rde-1*(*ne219*)*;viro-2*(*vir10*)	{*virIs1*[*PHIP*::OrsayRNA1;*PHIP*::OrsayRNA2; *Pmyo-2*::YFP]; *jyIs8*[*Ppals-5*::GFP; *Pmyo-2*::mCherry]; *rde-1*(*ne219*) V; *viro-2*(*vir10*) III}
WUM61	*virEx28*[*Psur-5*::*sid-3*::YFP; *Psur-5*::*viro-2*::mCherry]*;* *rde-1*	{*virEx28*[*Psur-5*::*sid-3*::YFP;*Psur-5*::*viro-2*::mCherry]; *rde-1*(*ne219*)V; }
WUM64	*sid-3*(*vir9*)*;virEx31*[*Psur-5*::*sid-3*]	{*virEx31*[*Psur-5*::*sid-3*;*Pmyo-3*::mCherry]; *jyIs8*[*Ppals-5*::GFP; *Pmyo-2*::mCherry]; *rde-1*(*ne219*)V; *sid-3*(*vir9*)X}
WUM65	*sid-3*(*vir9*);*virEx32*[*Psur-5*::*sid-3K139A*]	{*virEx32*[*Psur-5*::*sid-3K139A*;*Pmyo-3*::mCherry]; *jyIs8*[*Ppals-5*::GFP; *Pmyo-2*::mCherry]; *rde-1*(*ne219*)V; *sid-3*(*vir9*)X}
WUM66	*sid-3*(*vir9*); *virEx33*[*Psur-5*::*sid-3*::mCherry]	{*virEx33*[*Psur-5*::*sid-3*::mCherry;*Pmyo-2*::YFP]; *jyIs8*[*Ppals-5*::GFP; *Pmyo-2*::mCherry]; *rde-1*(*ne219*)V; *sid-3*(*vir9*)X}
WUM67	*sid-3*(*vir9*); *virEx34*[*Psur-5*::*sid-3K139A*::mCherry]	{*virEx34*[*Psur-5*::*sid-3K139A*::mCherry;*Pmyo-2*::YFP]; *jyIs8*[*Ppals-5*::GFP; *Pmyo-2*::mCherry]; *rde-1*(*ne219*)V; *sid-3*(*vir9*)X}
WUM68	*viro-2*(*vir10*);*virEx35*[*Psur-5*::*viro-2*]	{*virEx35*[*Psur-5*::*viro-2*;*Pmyo-3*::mCherry]; *jyIs8*[*Ppals-5*::GFP; *Pmyo-2*::mCherry]; *rde-1*(*ne219*)V; *viro-2*(*vir10*)III}
WUM69	*viro-2*(*gk627069*);*virEx36*[*Psur-5*::*viro-2*::mCherry]	{*virEx36*[*Psur-5*::*viro-2*::mCherry;*Pmyo-2*::YFP]; *jyIs8*[*Ppals-5*::GFP; *Pmyo-2*::mCherry]; *rde-1*(*ne219*)V; *viro-2*(gk627069)III}
WUM71	*sid-3*(*vir9*)*; viro-2*(*vir10*)	{*jyIs8*[*Ppals-5*::GFP; *Pmyo-2*::mCherry]; *rde-1*(*ne219*) V; *sid-3*(vir9)X; *viro-2*(*vir10*)III}
WUM72	*nck-1*(*ok694*)	{*jyIs8*[*Ppals-5*::GFP; *Pmyo-2*::mCherry]; *rde-1*(*ne219*) V; *nck-1*(*ok694*)X}

### Orsay virus preparation, infection, and RNA extraction.

Orsay virus was prepared by large-scale liquid culture as described previously (4), filtered through a 0.22-μm filter, and stored at −80°C. For all infection experiments, animals were bleached and then synchronized in M9 buffer (44) in 15-ml conical tubes with constant rotation at room temperature for 16 h. Five hundred arrested larval stage 1 (L1) larvae were seeded onto each well of a six-well plate with 20 μl *E. coli* OP50 lawn. L1 larvae were allowed to recover for 20 h at 20°C prior to infection. Orsay virus filtrate was thawed in a 37°C water bath and then diluted 1:10 with M9 buffer. For each well, 20 μl of virus filtrate was added into the middle of the bacterial lawn and incubated at 20°C. Three days after infection, animals were collected into 1.5-ml Eppendorf tubes by washing each well with 1 ml of M9 buffer and then pelleted by spinning for 1 min at 3,000 rpm in a benchtop centrifuge. M9 supernatant was removed, and 350 μl TRIzol reagent (Invitrogen) was added to the tubes, and then the tubes were frozen in liquid nitrogen. For each experiment, three replicate wells were used for each infection condition. Total RNA from infected animals was extracted using Direct-zol RNA miniprep (Zymo Research) purification according to the manufacturer’s protocol and eluted into 30 μl of RNase/DNase-free water.

### *C. elegans* feeding RNAi knockdown.

RNAi feeding was used for gene knockdown as described previously (45). *E. coli* strain HT115 carrying double-strand RNA expression cassettes for genes of interest was induced using established conditions and then seeded into six-well NGM plates. *dpy-3*, *sid-3*, *adr-2*, *lst-4*, *dhs-7*, *mig-13*, *mig-2*, *wsp-1*, and *nck-1* RNAi clones were from the Ahringer RNAi library (46). To construct the *viro-2* feeding RNAi clone, a 1-kb genomic fragment of the gene was amplified by single animal PCR using primers HJ245 (TGCGGATCCCATCAAGACTAC) and HJ246 (TACGGATCCAAGGGTTGGTAGTGTTCCG). The PCR product was digested with BamHI, cloned into pL4440, and transformed in *E. coli* HT115. Twenty arrested L1 *drh-1* mutant or N2 animals were seeded into each well of a six-well plate. After 72 h of RNAi feeding, Orsay virus was added to the plates as described above. At 48 h postinfection (hpi) the infected *C. elegans* animals were collected, and 350 μl of TRIzol (Invitrogen) was added to each well for RNA extraction.

### Orsay virus quantification through real-time qRT-PCR.

RNA extracted from infected animals was subjected to one-step real-time quantitative reverse transcription-PCR (qRT-PCR) to quantify Orsay virus replication as previously described (27). Briefly, the extracted viral RNA was diluted 1:100, and 5 μl was used in a TaqMan fast virus one-step qRT-PCR with primers and probe Orsay_RNA2 that target the Orsay RNA2 segment. Absolute Orsay virus RNA2 copy number was determined by comparison to a standard curve generated using serial dilutions of Orsay virus RNA2 *in vitro* transcripts. Primers GW314 and GW315 and probe Orsay_RNA1 that target the Orsay virus RNA1 segment were used to quantify Orsay virus RNA1 abundance (4). To control for variation in the number of animals, Orsay virus RNA levels were normalized to an internal control gene *cdc-42*, levels of which are known to be constant throughout the *C. elegans* life cycle (47). The means and standard deviations for three replicate wells are shown for all experiments. Statistical testing was done by Student’s *t* test. Statistically significant comparisons are indicated as follows: *****, *P* < 0.00005; ****, *P* < 0.0005; ***, *P* < 0.005; **, *P* < 0.01; *, *P* < 0.05; NS (for not significant with *P* > 0.05).

### Forward genetic screen for mutants unable to induce GFP expression following Orsay virus infection.

The *jyIs8;rde-1* reporter strain was maintained on 10-cm plates seeded with 1 ml of *E. coli* OP50. Animals were grown to a stage with a high proportion of L4 larvae and were then collected by washing the plates with water. Animals were then treated with 50 mM of ethane methylsulfonate (EMS) for 4 h at 20°C with constant rotating. Animals were washed with M9 buffer and recovered on plates for 2 h. L4 P0 animals were transferred to a new NGM plate and were allowed to lay F1 eggs. F1 animals were washed away from plates after laying about 20 eggs for each animal. Hatched F2 animals were challenged with 800 μl Orsay virus filtrate per plate and screened for Viro mutants.

Two hermaphrodites of each mutant strain were backcrossed with eight males generated from the unmutagenized *jyIs8;rde-1* reporter strain. Their progenies were assessed for the Viro phenotype to confirm Mendelian genetics for these mutants. Similarly, Viro-1 and Viro-2 mutants were reciprocally crossed with each other to define the ability of their progeny to complement the Viro phenotype.

### Genetic mapping through F2 bulk segregant and CloudMap analysis.

Viro-1 and Viro-2 mutants were crossed with the WM27 *rde-1*(*ne219*) strain, and F1 progenies were chosen and transferred to six-well plates. After 3 days, the F2 progenies were challenged with Orsay virus. A total of 72 F2 Viro animals were picked off F1 plates and transferred to six-well plates. F2 plates were replicated after 1 day of laying eggs. The original F2 plates were challenged again with Orsay virus, and both virus-induced GFP fluorescence and Orsay virus RNA levels were assayed at 3 days postinfection. Wells that yielded exclusively Viro animals and that yielded low RNA levels from replicated F2 plates were pooled, and genomic DNA from pooled samples was extracted using a Qiagen genomic DNA preparation kit according to the manufacturer’s protocol. DNA libraries were prepared using NEB-Next library preparation kit (New England Biolabs) and then sequenced using Illumina Hiseq 2500. The raw sequence data were analyzed using the pipeline CloudMap for *C. elegans* gene mapping (16).

### Plasmid construction for gene overexpression.

To construct the *sid-3* expression plasmid (*Psur-5*::*sid-3*), the entire *sid-3* gene and 500 bp of its native 3′ untranslated region (3′ UTR) were amplified from fosmid WRM061cD03. The PCR product was digested by BamHI and SacII and was then ligated into the plasmid containing the *sur-5* promoter. To generate the *sid-3* catalytic mutant expression plasmid (*Psur-5*::*sid-3K139A*), site-directed mutagenesis was performed to introduce a lysine-to-alanine (K139A) mutation in the SID-3 kinase domain. C-terminal mCherry fusion constructs of both the wild-type SID-3 and K139A mutant were generated by overlapping PCR. *Psur-5*::*viro-2* and *Psur-5*::*viro-2*::mCherry were generated in an analogous fashion.

### Ectopic *trans*-complementation with plasmids encoding SID-3 and VIRO-2.

For rescue of Viro-1, a fosmid (WRM061cD03) or an expression plasmid containing the *sid-3* gene was injected into the mutant strain at a concentration of 50 ng/μl along with 5 ng/μl of *Pmyo-3*::mCherry as a transgenic marker and 50 ng/μl of DNA ladder (NEB) to help establish the array (48). For rescue of Viro-2, the genomic fragment of *viro-2* containing 3 kb upstream of the promoter region and 500 bp downstream of the 5′ UTR or a plasmid that expresses *viro-2* was injected at a concentration of 12.5 ng/μl along with 2.5 ng/μl of *Pmyo-3*::mCherry and 87.5 ng/μl of DNA ladder. The stable transgenic array containing F2 animals was selected, challenged with Orsay virus, and assayed for GFP fluorescence and virus replication by qRT-PCR. The expression levels of the virus-induced gene F26F2.1 in the *sid-3* rescue experiment were measured by a two-step real-time quantitative PCR using TaqMan gene expression assays (Applied Biosystems) according to the manufacturer’s protocol.

### Epifluorescence and confocal microscopy.

The microscopic visual scanning analysis for Orsay virus infection was carried out using a Leica stereo fluorescence microscope. Images of *C. elegans* mutant infections were acquired using a Zeiss Axioskop inverted fluorescence microscope. Briefly, young adult to adult *C. elegans* animals were anesthetized with 1 mM levimisol and then put on a 2% dry agarose pad with a coverslip (5 by 5 cm) on top. Images were acquired from both fluorescence channels and bright-field channels. Confocal images for colocalization of SID-3 and VIRO-2 were acquired using a Zeiss LSM 880 confocal microscope with Airyscan (Carl Zeiss, Inc., Thornwood, NY) equipped with a 63×, 1.4-numerical-aperture Zeiss Plan Apochromat oil objective. Quantification of mCherry expression was performed by image analysis with ImageJ 1.51j8.

10.1128/mBio.00940-17.9 FIG S9  Quantification of virus-induced gene F26F2.1 RNA levels. Real-time qPCR of Orsay virus-induced gene F26F2.1 expression levels after virus infection in *sid-3*(*vir9*) strain and with ectopic expression of SID-3. ***, *P* < 0.005; NS, not significant (*P* > 0.05). Download FIG S9, TIF file, 0.1 MB. Copyright © 2017 Jiang et al. 2017 Jiang et al. This content is distributed under the terms of the Creative Commons Attribution 4.0 International license.
