# Supplementary figures and images for: An Evolutionarily Conserved Pathway Essential for Orsay Virus Infection of Caenorhabditis elegans
Source: mBio. 2017 Sep 5;8(5):e00940-17. doi: 10.1128/mBio.00940-17 (PMC5587906; doi:10.1128/mBio.00940-17)

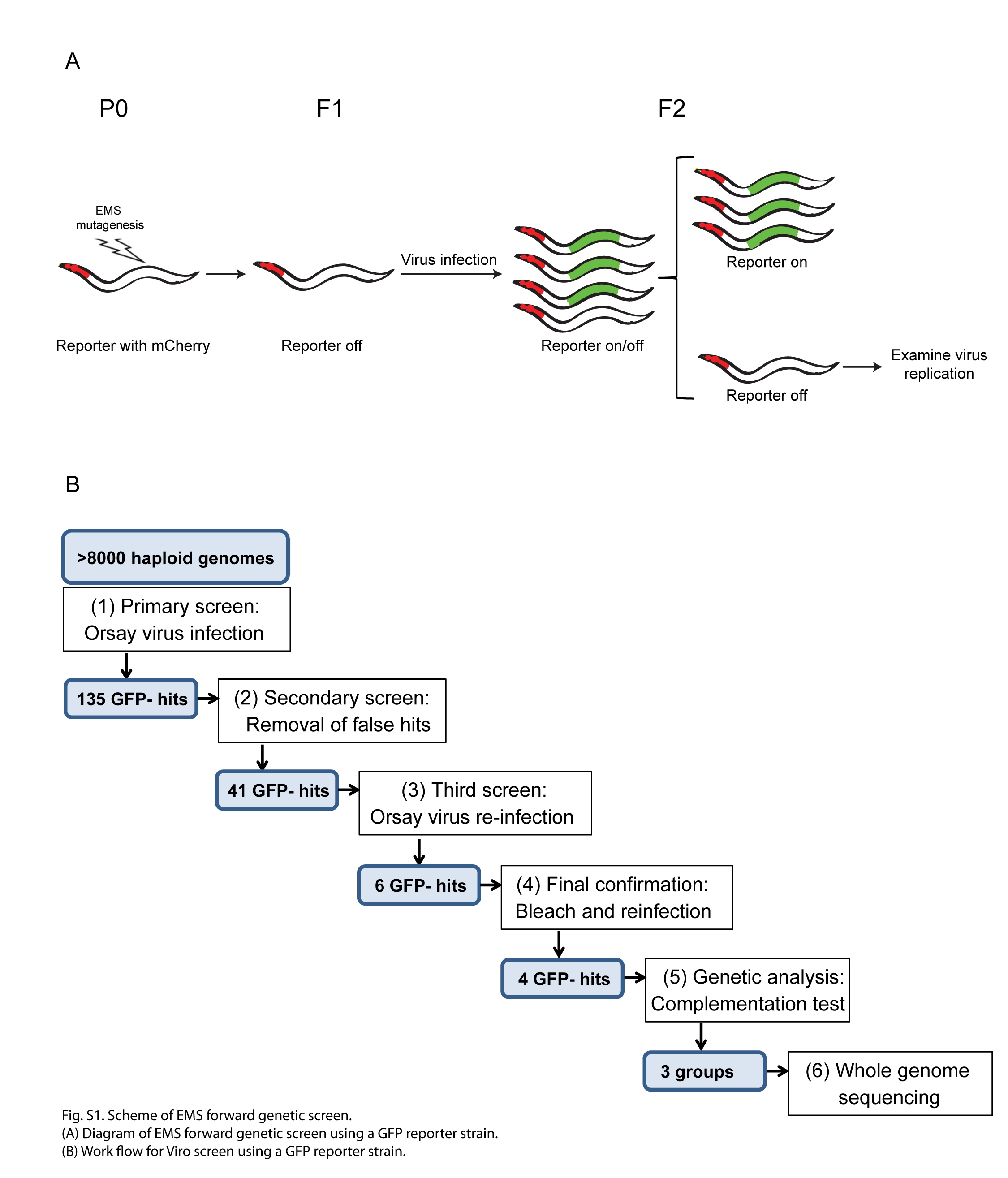

Supplement: FIG S1 [file mbo004173455sf1.tif]

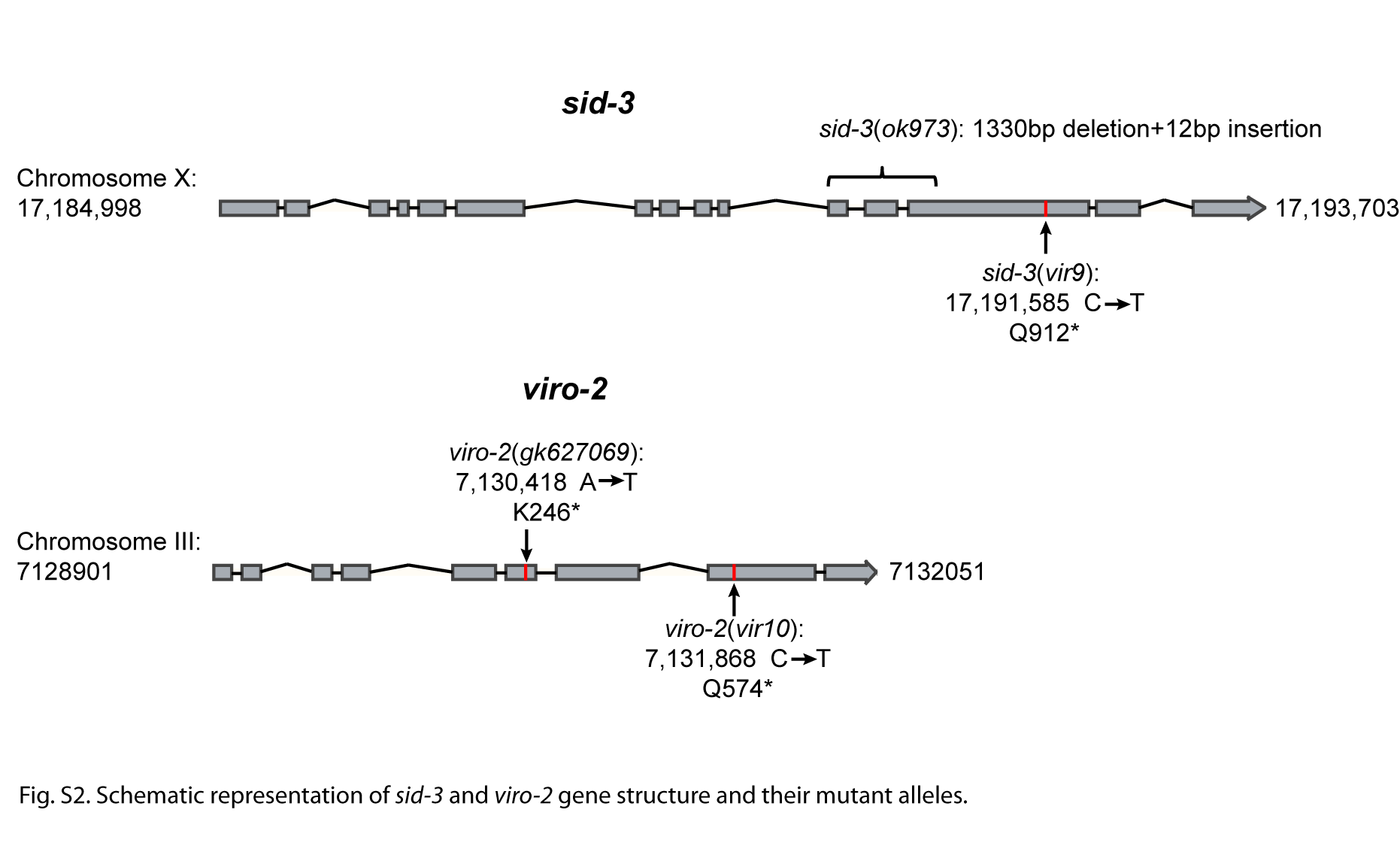

Supplement: FIG S2 [file mbo004173455sf2.tif]

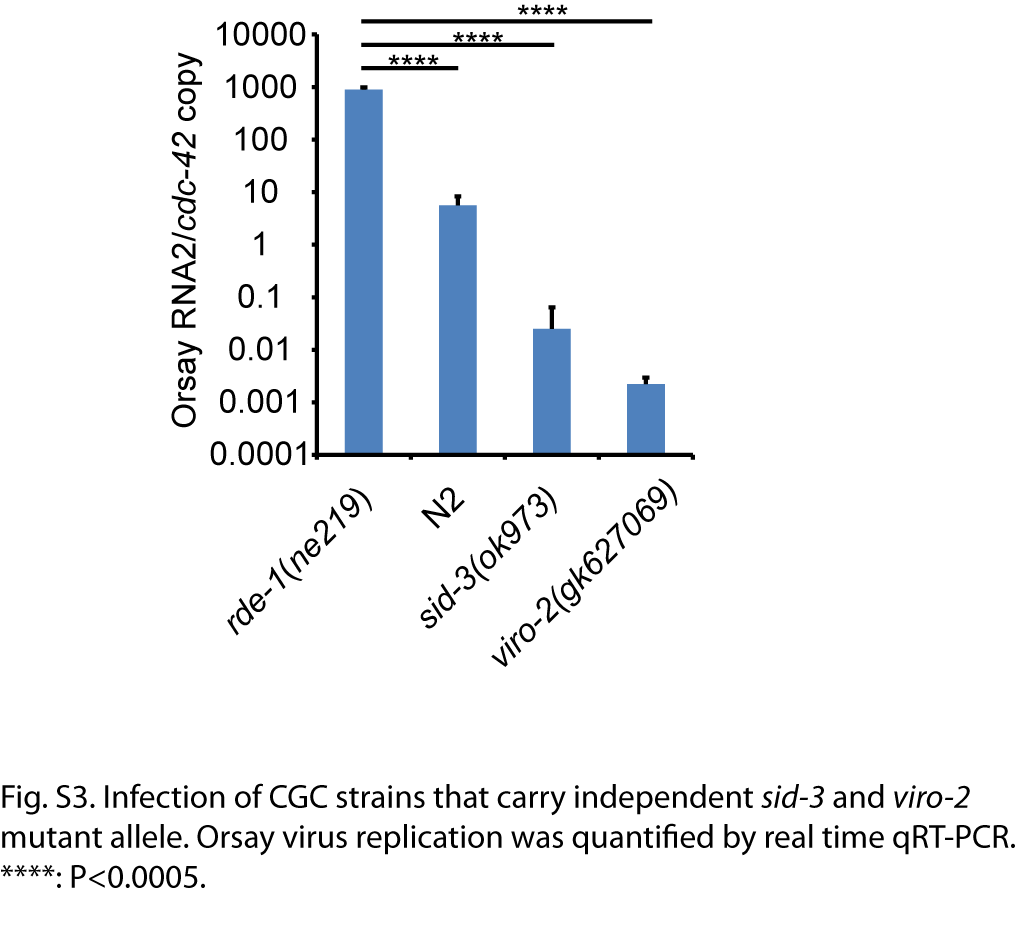

Supplement: FIG S3 [file mbo004173455sf3.tif]

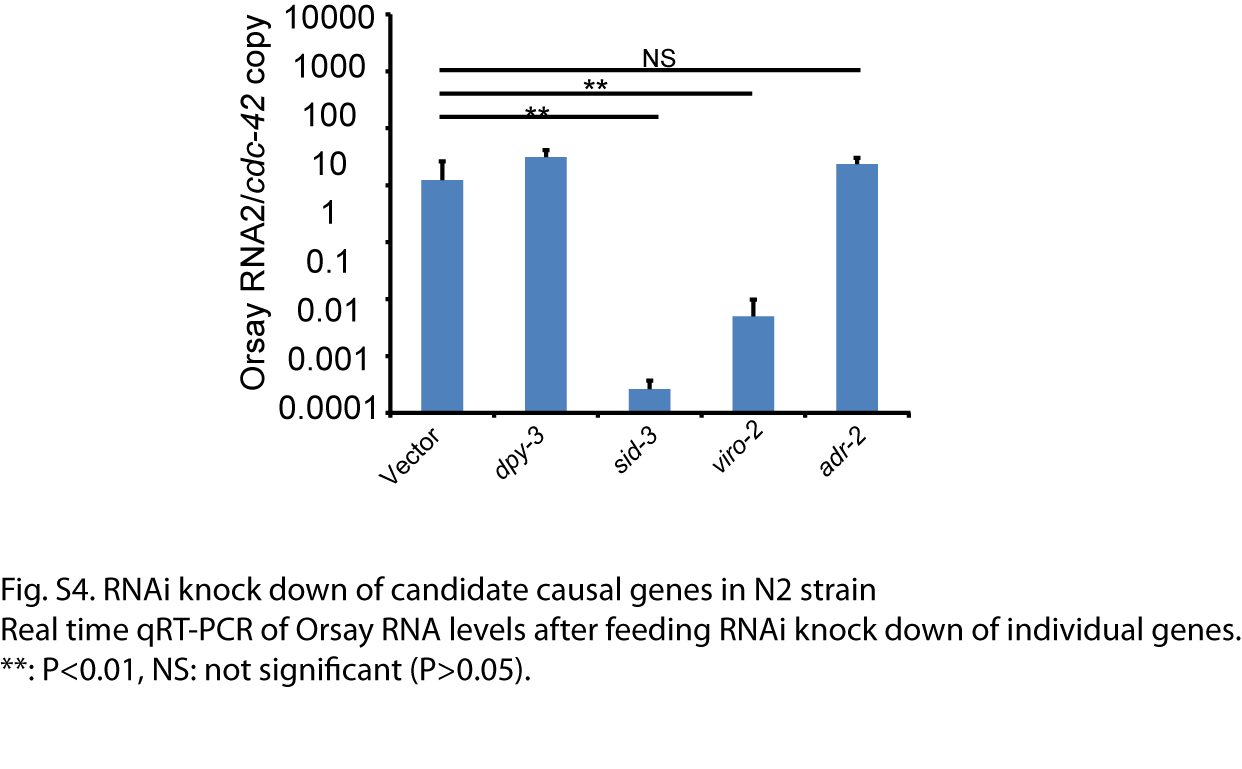

Supplement: FIG S4 [file mbo004173455sf4.tif]

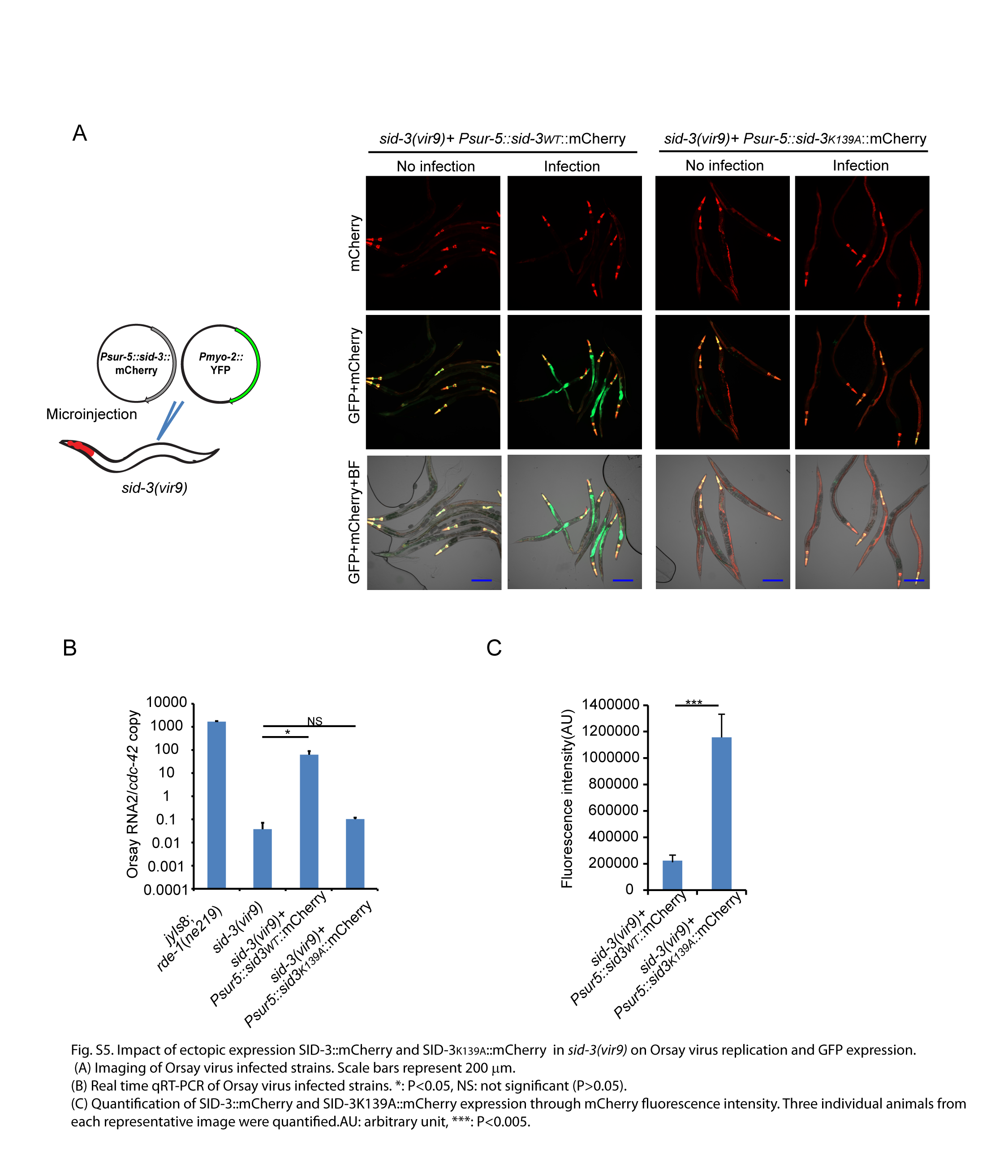

Supplement: FIG S5 [file mbo004173455sf5.tif]

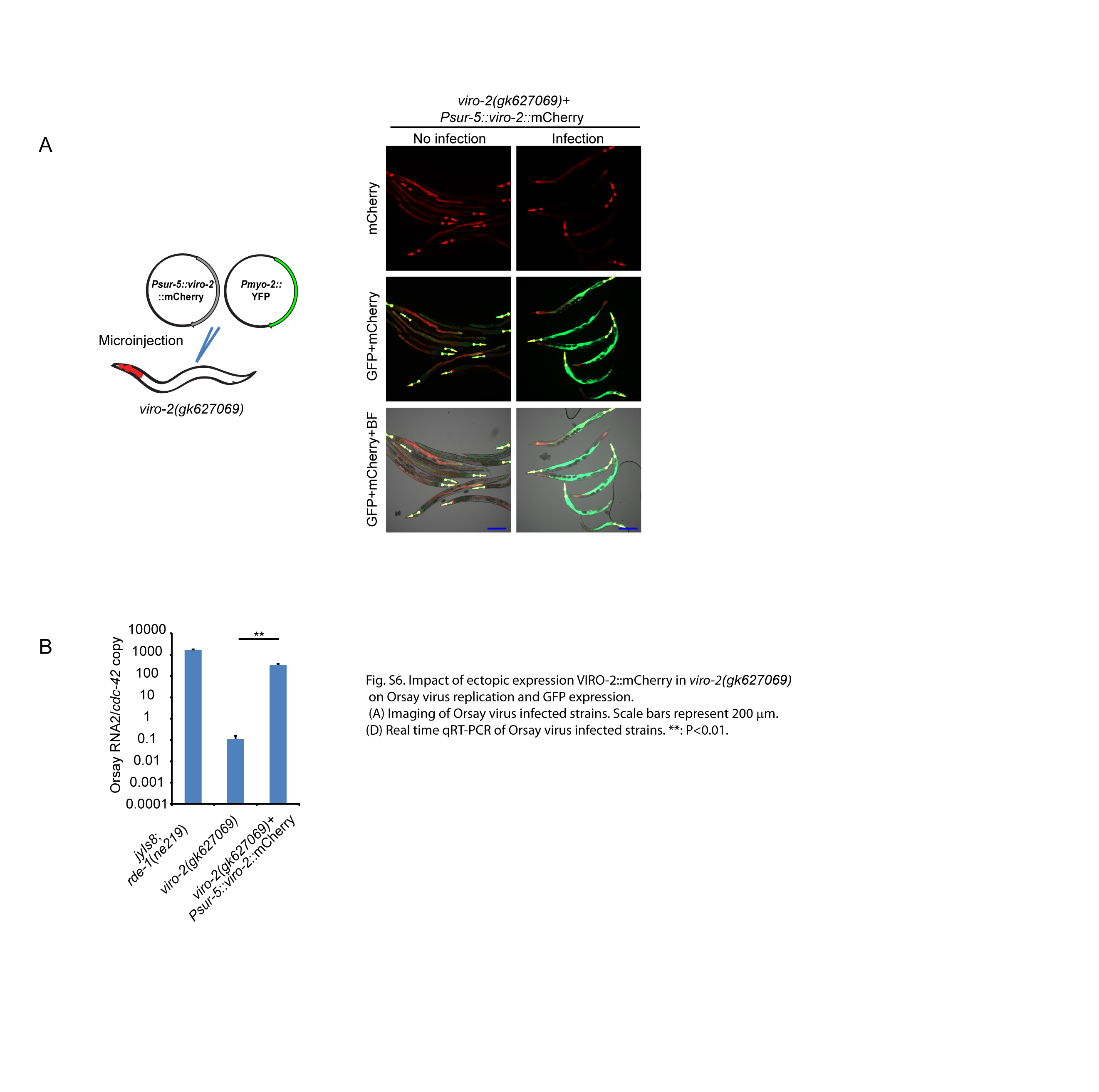

Supplement: FIG S6 [file mbo004173455sf6.tif]

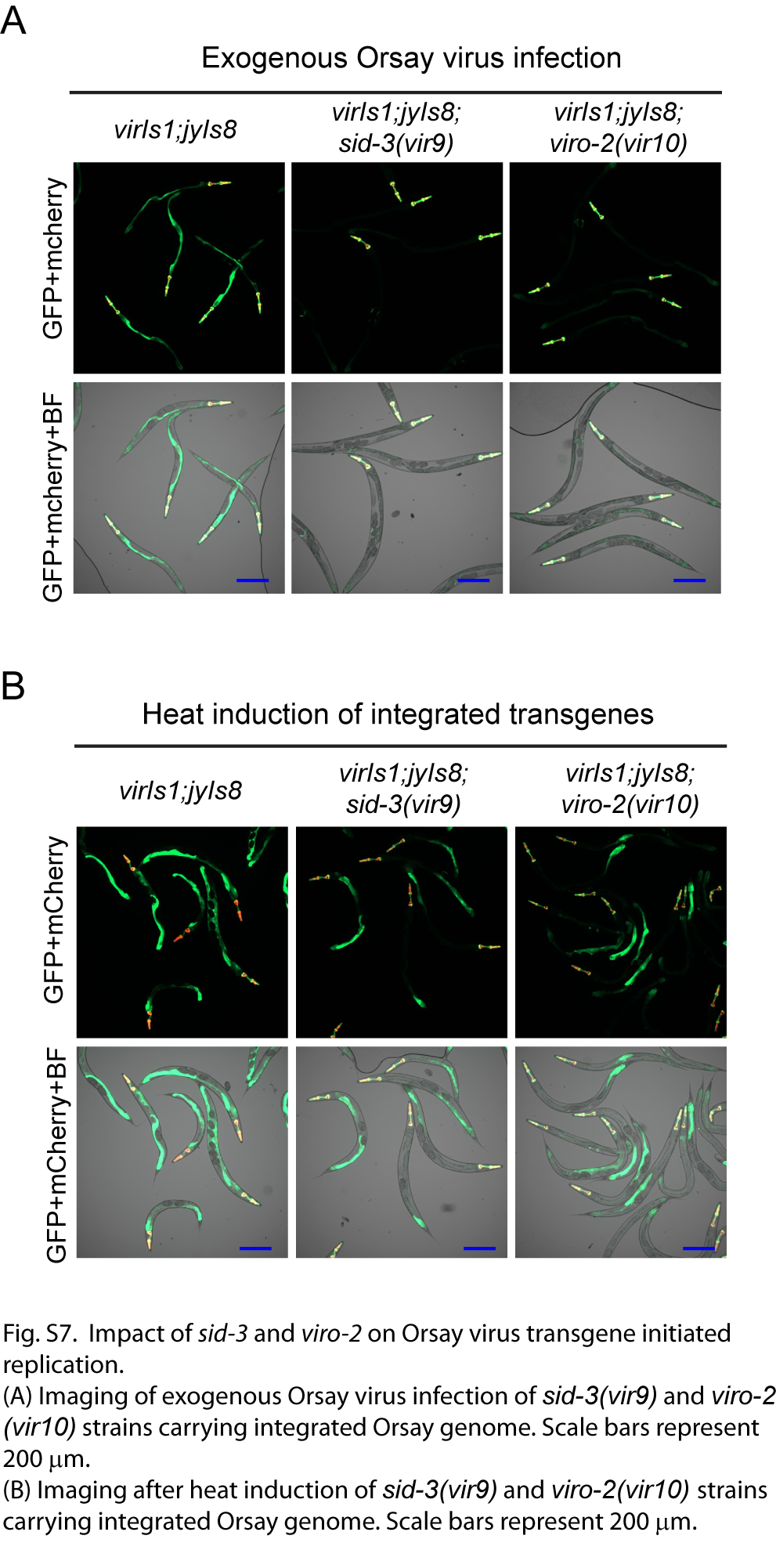

Supplement: FIG S7 [file mbo004173455sf7.tif]

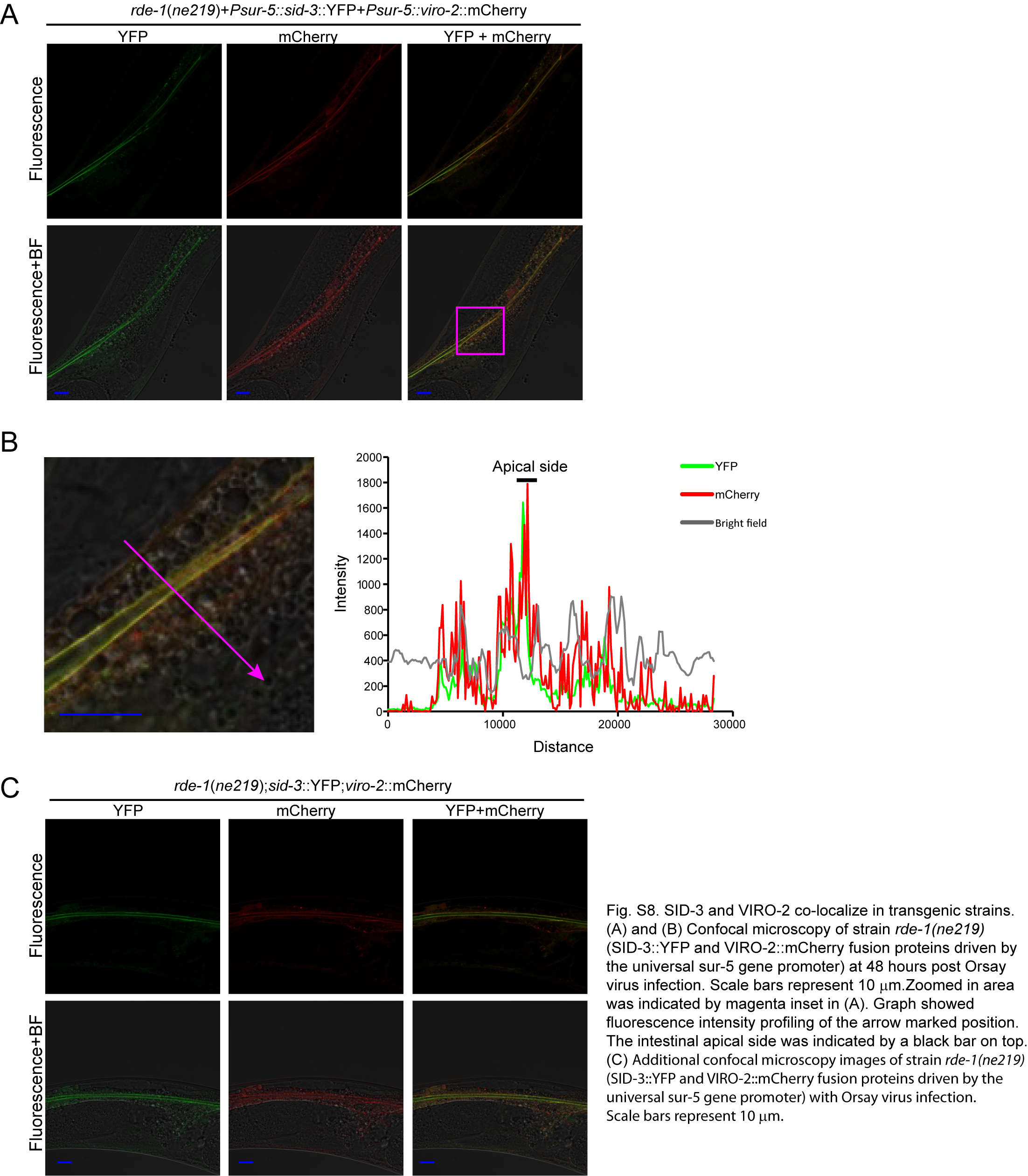

Supplement: FIG S8 [file mbo004173455sf8.tif]

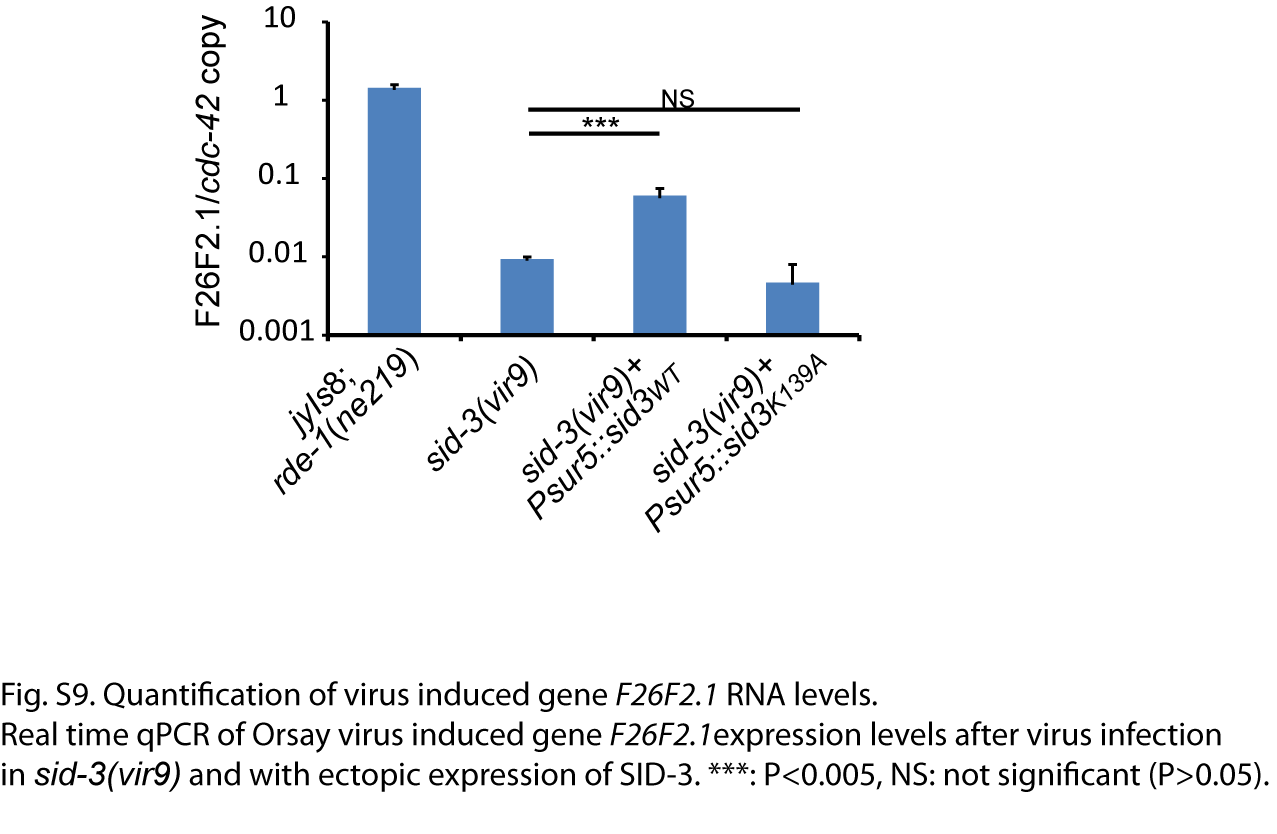

Supplement: FIG S9 [file mbo004173455sf9.tif]
